# Reforming the Chimeric Antigen Receptor by Peptide Towards Optimized CAR T Cells With Enhanced Anti-Cancer Potency and Safety

**DOI:** 10.3389/fbioe.2022.928169

**Published:** 2022-06-17

**Authors:** Cuijuan Liu, Lin Li, Fan Gao, Jundong Zhou, Yingzhou Qin, Xin Yuan, Guang Yang, Yimin Zhu

**Affiliations:** ^1^ School of Nano Technology and Nano Bionics, University of Science and Technology of China, Hefei, China; ^2^ CAS Key Laboratory of Nano-Bio Interface, Suzhou Institute of Nano-Tech and Nano-Bionics, Chinese Academy of Sciences, Suzhou, China; ^3^ Nanjing Medical University, Affiliated Suzhou Hospital, Department Radio Oncology, Suzhou, China; ^4^ Department of Oncology, Suzhou BenQ Medical Center, The Affiliated BenQ Hospital of Nanjing Medical University, Suzhou, China

**Keywords:** chimeric antigen receptor, off-tumor effect, peptide, anti-cancer therapy, affinity

## Abstract

The emerging chimeric antigen receptor (CAR) T cell revolutionized the clinic treatment of hematological cancers, but meet its Waterloo in solid tumor therapy. Although there exist many reasons for this limitation, one of the largest challenges is the scarcity of recognition for tumor cells, resulting in the undesirable side effects and the subsequent ineffectiveness. To overcome it, a lung-cancer-cell-targeting peptide termed A1 was used in this work to reform the scFv domain of CAR by genetic manipulation. As a result, this modified ^A1^CAR T exhibited the optimized cancer-cell targeting and cytotoxicity *in vitro* and *in vivo*. More importantly, by tuning the sensitivity of CAR to antigen, peptide-based ^A1^CAR T cells could distinguish tumors from normal tissue, thereby eliminating the off-tumor toxicity in healthy organs. Collectively, we herein constructed a genetic peptide-engineered CAR T cells by inserting A1 peptide into the scFv domain. Profitted from the optimized recognition pattern and sensitivity, ^A1^CAR T cells showed the ascendancy in solid tumor treatment. Our findings demonstrate that peptide-based CAR T holds great potential in solid tumor therapy due to an excellent targeting ability towards tumor cells.

## Introduction

In 2020, 10 million of the 19.3 million people diagnosed with cancer have died of cancer. (Sung et al., 2021). The high fatality rate highlights the urgent requirement to develop effective anti-cancer treatments. Towards this end, Immunotherapy is a rapidly growing area that utilizes the immune system’s potential to eliminate tumors, and chimeric antigen receptor T cells (CARs) have powerful anticancer efficacy. (Grupp et al., 2013; Gun et al., 2019). CAR is a fusion protein composed of an antigen recognition moiety (e.g., antibody single chain variable fragments, scFv) and T cell self-activation signaling moiety (Maher et al., 2002; Sadelain et al., 2013; 2016). The FDA has approved five CARs products to treat hematologic malignancies. (Schuster et al., 2017; DiNofia and Maude, 2019). Selecting tumor specific antigen (TSA) as targets provides a way forward to significantly reform the security of CARs (Sadelain et al., 2013; Huang et al., 2020). Nonetheless, cancers barely show unique antigenic markers (Yong et al., 2017; Qu et al., 2021; Xiao et al., 2021; Chen et al., 2022). Most antigens are expressed in possibly vital organ tissues, which are called tumor associated antigens (TAA), such as MUC1 (mucoprotein 1), EGFR (epidermal growth factor receptor), ErbB2 (HER2, CD340), GD2 (disialoganglioside), and PSMA (prostate-specific membrane antigen) (MacKay et al., 2020). The specific targeting of these antigens by CARs is limited by normal tissue toxicity (Parkhurst et al., 2011; Brudno and Kochenderfer, 2019). To be sure, ErbB2-based designated CARs therapy intended to treating malignant colon cancer were demonstrated deadly to patient, largely because expression of TAA on lung epithelial cells (Morgan et al., 2010). The basical expression levels of EGFR in skin tissue prompts dose-limiting skin harmfulness (Lamers et al., 2013). Therefore, CARs targeting to TAA would induce the on-target, off-tumor toxicity to human organs (Chmielewski et al., 2004; Morgan et al., 2010; Corse et al., 2011; Parkhurst et al., 2011; Hudecek et al., 2013; Lamers et al., 2013; Brudno and Kochenderfer, 2019). Targeting molecules with low affinity to TAA may provide a way forward for design of CARs in treating malignant solid tumors.

The decision of single chain spacer (Milone et al., 2009), extracellular and costimulatory domains in the design of CAR plasmid significantly affect CARs capacity and performance. Be that as it may, little is had some significant awareness of the impact of CAR binding affinity. It has been reported that increasing affinity between receptor and antigen beyond a certain point may adversely affect T cell responses (Schmid et al., 2010; Tan et al., 2015; Richman et al., 2018). T cell’s activation may require the accumulated stimulation from a few high-affinity or large number of low-affinity TCRs (Thomas et al., 2011; Tran et al., 2013; Brudno and Kochenderfer, 2019). Previous work from Chmielewski *etc.* (Thomas et al., 2011; Tran et al., 2013; Brudno and Kochenderfer, 2019; Ghorashian et al., 2019) proposed that high-affinity CARs showed less separation between target cells with high or low antigen expression levels. By increasing the KD (reduced affinity) 2- to 3-log of scFv used in CARs, a significant enhancement was accomplished within the restorative list for ErbB2 and EGFR CARs (Caruso et al., 2015; Liu et al., 2015). Additionally, Pameijer, C. R. J. *et al.* discovered that the scFv could be substituted with peptide ligand in CARs therapy (Pameijer et al., 2007; Whilding et al., 2017; Wang et al., 2020). With the lower affinity antigen receptor than scFv, peptide-based CARs would be activated only when they were docked with overexpressed TAA on tumor cells but not with low, baseline TAA expression on normal cells (Arcangeli et al., 2017; Drent et al., 2017; Walker et al., 2017; Drent et al., 2019; Di Roberto et al., 2020).

The CAR’s scFv can be substituted with a peptide ligand that interacts with tumor-overexpressed receptors. Chimeric NKG2D receptors (Zhang et al., 2006; Deng et al., 2019; Frazao et al., 2019), IL-13-cytokine CARs for IL-13R2-expressing tumor cells (Kahlon et al., 2004; Sengupta et al., 2014; Brown et al., 2016), integrin v6-binding peptides (Pameijer et al., 2007; Whilding et al., 2017), and heregulin-chimaeras are all presently in preclinical and early clinical trials. Such peptide-based chimeric antigen receptors are proved less immunogenic than traditional scFvs, since they are human protein and are hence liable to be perceived as self-proteins. Peptides with good binding ability have low molecular weights and it is feasible to link peptides in tandem repeats in one molecule. To evaluate performance of peptides in a CAR format, we designed a peptide-based CAR using peptide A1 (WFCSWYGGDTCVQ). Peptide A1 was discovered and identified to specifically bind to the human lung carcinoma A549 cells (Dong et al., 2013). We integrated the peptide in CAR designs and surveyed the antitumor capacity of CARs. *In vitro* and *in vivo,* we demonstrated that the peptide CARs did not compromise the anti-tumor efficacy and improved their immunotherapeutic potential.

## Materials and Methods

### Peptide-CAR Lentiviral Design and Construction

The CAR constructs are contained in a Lentiviral vector under control of hEF1-α promoter, the Lentiviral vector, which was a gift from the Icartab Biomed of Suzhou. Cloning was done in Stbl 3 *E. coli* cells. To produce virus, HEK 293T cells (the human embryo kidney cells) are dealt with PEI (sigma) with the second generation Lentiviral CAR vector, a pSPAX2 and a pMD 2.0 G packaging vector.

The sequence of A1-CAR is as follows: A1 peptide (WFCSWYGGDTCVQ), linker (GGSGGQ), c-Myc tag (CAE84874, aa 1–11), CD8 (transmembrane and cytosolic, NP_001759, aa 167–235), 4-1BB costimulatory signal (AAA53133, aa 214–255) and CD3-ζ (cytosolic, NP_932170.1, aa 52–164). Scramble-CAR sequence is identical to A1-CAR, with the exception of the scramble peptide (DCQYFWSCGGWVT). Cetux-CAR sequence: the cetuximab light chain (PDB:1YY9_C, aa 1–213), Whitlow linker (AAE377080.1, aa 1–18), and cetuximab heavy chain (PDB:1YY9_D, aa 1–221).

### Reagents and Cell Culture

Human recombinant protein IL-2 (cat. #11848-HNAY1) were purchased from BD Biosciences. Human T cell-activated CD3/CD28 beads (cat. #11130D) and FITC-labeled human EGFR protein (cat. No. EGR-HF2H5) were purchased from ACRO Biosystems. FBS (cat. #SV30087.02), RPMI1640 medium (cat. #SN30809.06), and penicillin-streptomycin solution (cat. #RF67729.18) were obtained from HyClone. Anti-IFN-γ (cat. #BS9841) was purchased from BOSTER. Human Interleukin-13 ELISA Kit (EK1162) were purchased from sabbiotech. Anti-GZMB (granzyme B) (cat. #24699-2-SO) was purchased from Proteintech. Antihuman CD3 (cat. #17-9930-60) was purchased from eBioscience. D-Luciferin potassium salt (cat. #M8873) was bought from AbMole. Anti-CD31 (cat. #ab37167-050) and IFN-γ ELISA Kit (cat. #70-EK1802) was obtained from Multisciences. CD3^−^CD28 Dynabeads were purchased from Life Technologies.

The human renal carcinoma cell line ACHN, 786-o and the non-small human lung cancer cell A549 cell line, and MCF-7 cell line were purchased from the Chinese Academy of Sciences cell bank, which were verified by short pair rehash composing strategy. The MDB-MB-231 and HEK-293T was a gift from Prof. Guangli Suo (Chinese Academy of Sciences). All tumor cells are growing in5% CO2 at 37°C in the Thermo CO_2_ incubator.

### T Cell Isolation, Activation and CAR Transduction and Generation

1.5 × 10 ^7^ peripheral blood mononuclear cells (PBMC) were segregated from the human blood according to the producer’s guidelines. To generate CARs, T cells were segregated from PBMC by flow cytometry (BD Aria II)following anti-CD3 labeled. Lymphocytes were transduced with lentivirus relating to different second generation CARs develops. Briefly, the human T cells were enacted on day 0 with against human CD3/CD28 Dynabeads and cultured in T cell culture medium with 10% FBS and 20 IU ml^−1^ recombinant protein human IL-2. The activated human T cells were transduced using the Lentivirus generated from 293 T cells with the Lentiviral CAR vector, a pSPAX2 and a pMD 2.0 G packaging vector with PEI (1 mg/ml) on day 3. The activated human T cells were spined at 2000 g with the Lentivirus and polybrene (7.5 ng/ml) mixture. After two spin-fections, cells were allowed to grow until day 10 and along these lines moved to capacity in fluid nitrogen before functional assays. For every practical examine, all kinds of CARs were obtained from the one human. They were all under the same conditions to expansion. In specific analyses CARs were sorted on BD FACS Aria to acquire a pure population of transduced, c-Myc positive T cells on day 10. The number of CARs was calculated using absolute counter tube by flow cytometry based on beads.

### Characterization of Peptide Surface Display and Binding Assays

The positive rate of the CARs was portrayed utilizing a flow cytometry based assay. For most CARs, the positive rate was stained with c-Myc antibody to evaluate binding to effectively transduced cells. Briefly, this experiment was carried out at 4°C and cells were prepared in PBS buffer. The CARs was incubated with c-Myc antibody at 4°C for 15 min. Then the binding capacity of CARs was estimated with anti-c-Myc antibody.

### *In Vitro* Cytotoxicity and Activation Assays

CARs’s cytotoxicity was appeared utilizing different CARs with target cells co-culture measures. For cytotoxicity, cocultured the CARs with A549-luc cells overnight and supernatants were gathered and utilized for IL-2 and IFN-γ ELISA estimations. The A549-luc cells (human lung cancer cells with stable luciferase express) expressed EGFR naturally. A549-luc cells were cultured with RPMI 1640, and A1-CARs were included with the A549-luc cells for 18 h hatching. Then add D-Luciferin potassium (15 mg/ml) into the supernatants and the number of lived A549 cells were measured with Cytation 3. Results were analyzed based on luciferase activity: % killing = [RLU (relative light units) of control group - RLU of test group]/(RLU of control group) × 100. Supernatants of verious group were gathered to be utilized of IFN-γ estimations (Multisciences). For proliferation assays, PBMCs and CARs were expanded and then sorted on c-Myc-positive CARs.

### *In Vivo* Studies

Female nude mice (Cavens) aged 5 weeks were raised in good environmental conditions, which is specific pathogen-free (SPF) conditions. The ethical approval number for all animal experiments is SINANO/EC/2019–013 approved by the local Ethical Committee for Animal Experiments.

The A549 cells were screened by adding with puromycin (1 mg/ml) and sorted by flow cytometry after transfect with the plvx-puro/luciferase lentiviral vector.

For xenograft tumor studies, nude mice were given s. c. injection with A549 cells (1×10^6^) suspended in PBS. For CARs treatment, mice were given i. p. injection with 200 mg/kg cyclophosphamide for depleting circulating lymphocytes on the fourth day (Li et al., 2017). After 6 days, 5 × 10^6^ of scFv-T cells or peptide-based CARs were given i. v. injection on days 10 and 17.

The size of the tumor volume was measured through the bioluminescence by photoing with the IVIS Lumina II system (PerkinElmer). A total of five measurements over a 51-day period, one every 6 days. Before all the mice were sacrificed on day 51, collecting various organs and all tumor tissues. The dimensions of tumor were measured with calipers, and the volume of tumor was figured: V = 1/2 ab^2^, where a and b represented the tumor length and width, respectively.

### Immunohistochemistry

The articulation human IFN-γ and GZMB in cancer tissues of each not set in stone by IHC with comparing antibodies. Advanced images were taken by a Zeiss Scope A1, and the stained region of immuno-positive level was surveyed by the computerized picture breaking down programming ImageJ.

### Statistical Analysis

All the data were expressed as means ± SD. Histograms and line charts were generated by GraphPad Prism 5.0. T tests were utilized to decide the *p* values. *p < 0.05, **p < 0.01, ***p < 0.001, ****p < 0.0001.

## Results

### Design and Characteristics of Peptide-Based CARs

**FIGURE 1:**
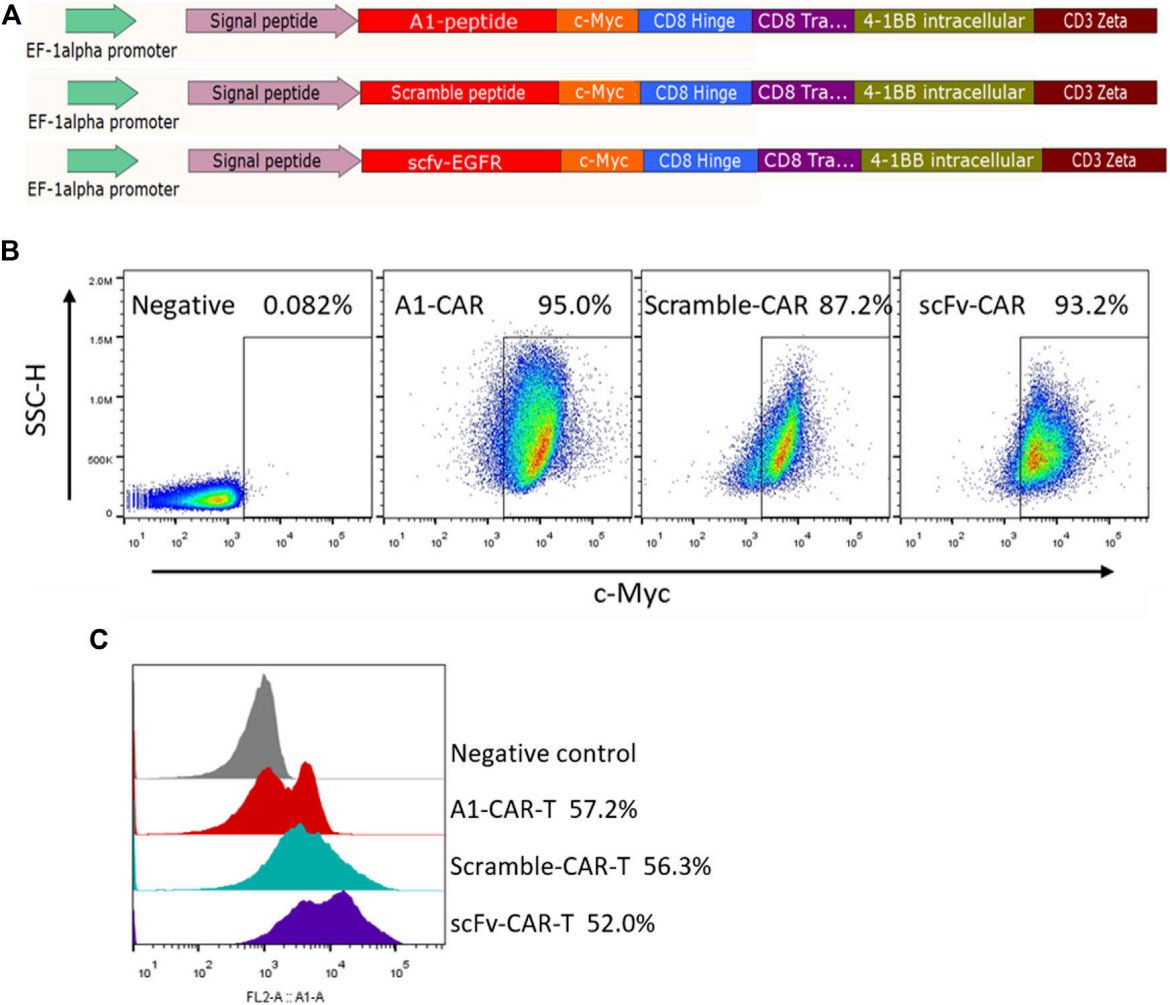
Design and characteristics of peptide-based CARs. **(A)** Lentiviral constructs of peptide-based A1-CAR, Scramble-CAR and EGFR-scFv-CAR. **(B)** Surface expression of the CAR on Jurkat cells at the end of the primary expansion on day 3 detected by binding to c-Myc antibody. Representative of three donors. **(C)** Surface expression of the CAR on the human T cells at the end of the primary expansion on day 3 detected by binding to c-Myc antibody. Representative of three donors.

In this study, peptide-based CARs were developed following the main design of single-chain antibody CARs, in which the peptide replaced the scFv as the recognition module. Peptides are much shorter than scFvs and it is feasible to link peptides in tandem repeats in one molecule. For the construction of these CARs, we employed the A1 peptide (WFCSWYGGDTCVQ) specific for A549 cells (Dong et al., 2013; Liu et al., 2019). For most experiments, a scramble peptide (DCQYFWSCGGWVT) was designed and served as the negative (nonspecific) control (Li et al., 2018), and Cetuximab scFv was chosen as the positive control. We named them A1-CAR, scramble-CAR and scFv-CAR, respectively. (Figure 1A). We tested the recognition ability of the peptide CAR by developing CAR-Jurkat (Figure 1B). Before developed CARs with Lentivirus, T cells sorted from human PBMC were activated with CD3/CD28 Dynabeads for 3 days. T cells were successfully transduced, and the positive rate of peptide-based CARs was almost comparable to or lightly higher than scFv-CARs (c-Myc-positive, typically 40–80% transduced) (Figure 1C). As can be seen from a FACS-based assay, the A1-peptide CAR successfully transduced into T cells. These results reveal that peptide is easily displayed as CAR recognition modules on human T cells.

### Peptide Based CARs Could be Effectively Activated and Showed Killing Effect on A549 Tumor Cells in Vitro

**FIGURE 2:**
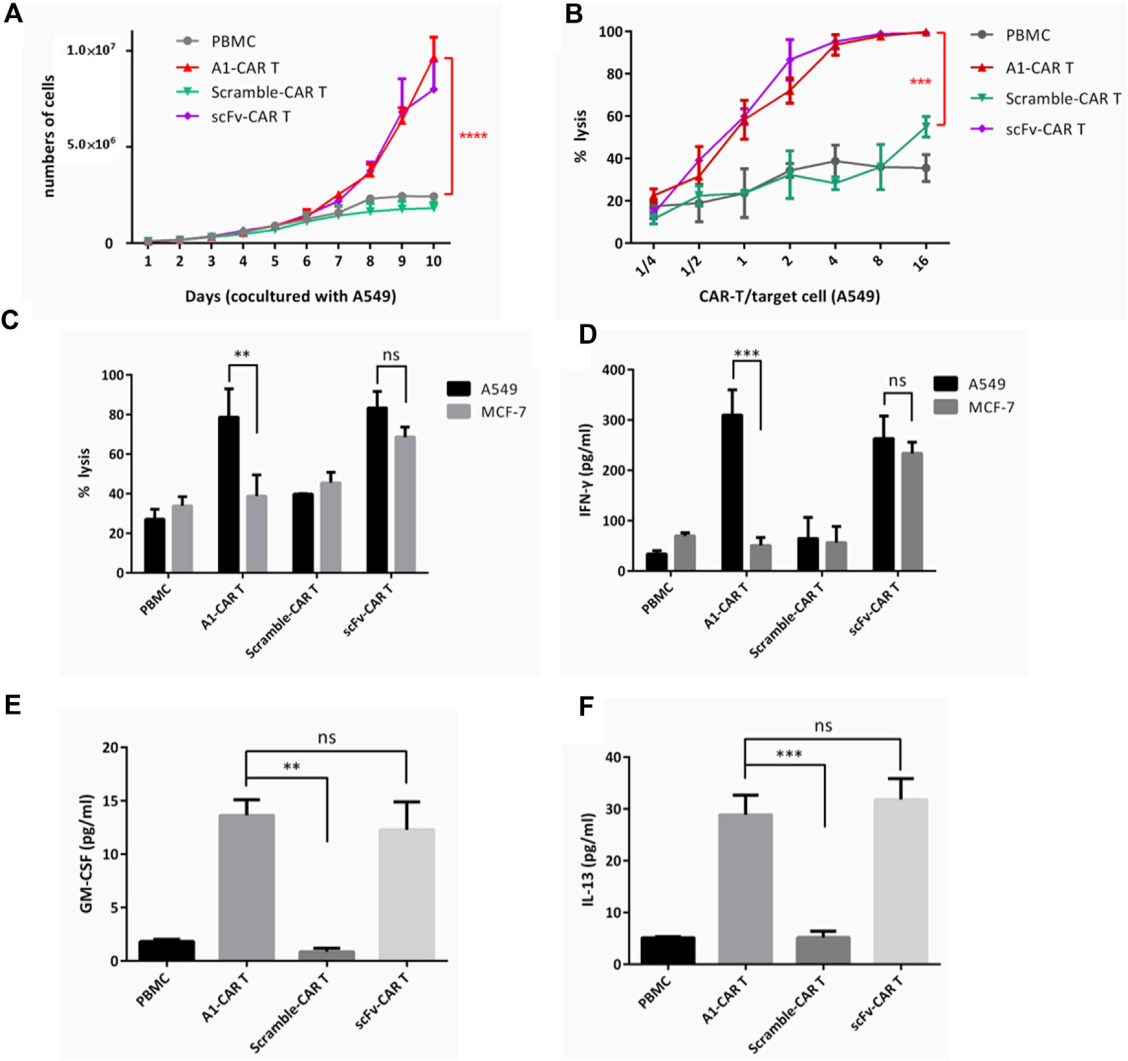
Peptide based CARs could be effectively activated and showed killing effect on A549 tumor cells *in vitro*. **(A)** On different days 1:1 cocultured with mitomycin-C-treated A549 cells, the numbers of PBMC, A1-CARs, scFv-CARs, and Scramble-CARs were examined (n = 3). T tests were utilized to decide the *p* values. **(B)** Cytotoxicity assay using A549-luc cells as targets. Data represent the mean ± SD of quadruplicate wells. T tests were utilized to decide the *p* values. **(C)** CARs were blended with target cells (A549-luc) at the effector/target (E/T = 4:1) ratios. 24 h later, Cytation three was used to detect bioluminescence to evaluate the percentage of lysis. CARs generated from six individual human donors. T tests were utilized to decide the *p* values. **(D–F)** CARs were blended with target cells (A549-luc) at the effector/target (E/T = 4:1) ratios. Supernatants were obtained 24 h after coculture. The amount of IFN-γ, GM-CSF and IL-13 was analyzed by fluorescence-based ELISA Kit (*n* = 4). Data represent the mean ± SD. *T* tests were utilized to decide the *p* values.

We firstly assessed the function of A1 peptide on the CARs. As higher expression of CAR is known to upgrade the proliferation and lysis target cell properties of CARs (Sadelain et al., 2013; 2016), the absolute number of cell divisions was evaluated. Multiple rounds stimulation by co-culturing with mitomycin-C-treated A549 cells, induced persistent cellular expansion of A1-CARs. The number was slightly higher for peptide-based CARs compared to traditional scFv-CARs (Figure 2A). Having appeared the authoritative specificity of peptide CARs, we decided to investigated functional properties of peptide-based CARs, such as targeting killing effect and the function of cytokine release. To investigate if A1-CARs targeting A549 cells could specifically recognize and lyse A549 cancer cells, a bioluminescence-based cytolytic assay was settled using the human lung carcinoma A549-luc cells. As demonstrated in Figure 2B, The L1-CARs lysed the A549-luc cells in a dose-dependent way. No noteworthy differences in cytotoxicity were observed between peptide-based CARs and scFv-CARs (Figure 2C). Meanwhile, the function of cytokine release of A1-CARs following co-cultured with A549 lung cancer cell was assessed. Upon incubation of A1-CARs with A549 cells, there were great increases in IFN-γ, GM-CSF and IL-3 in the culture supernatants of A549 cells-specific CARs compared to negative (nonspecific) control (Figures 2D–F). These results reveal that the kill ability of the A1 peptide-CARs to A549 tumor cells was specific. Specific cytotoxicity appeared in a dose-dependent way. That came to the conclusion that the peptide-based CARs have great specific cytotoxicity to tumor cells.

### A1-CARs Are More Effective in Inhibiting the Growth of Xenograft Tumors

The superior killing ability of our A1-CARs target A549 cells prompted us to further investigate its tumor-killing properties *in vivo*. The antitumor activities of A1-CARs *in vivo* were assessed in the xenograft mouse model.

**FIGURE 3:**
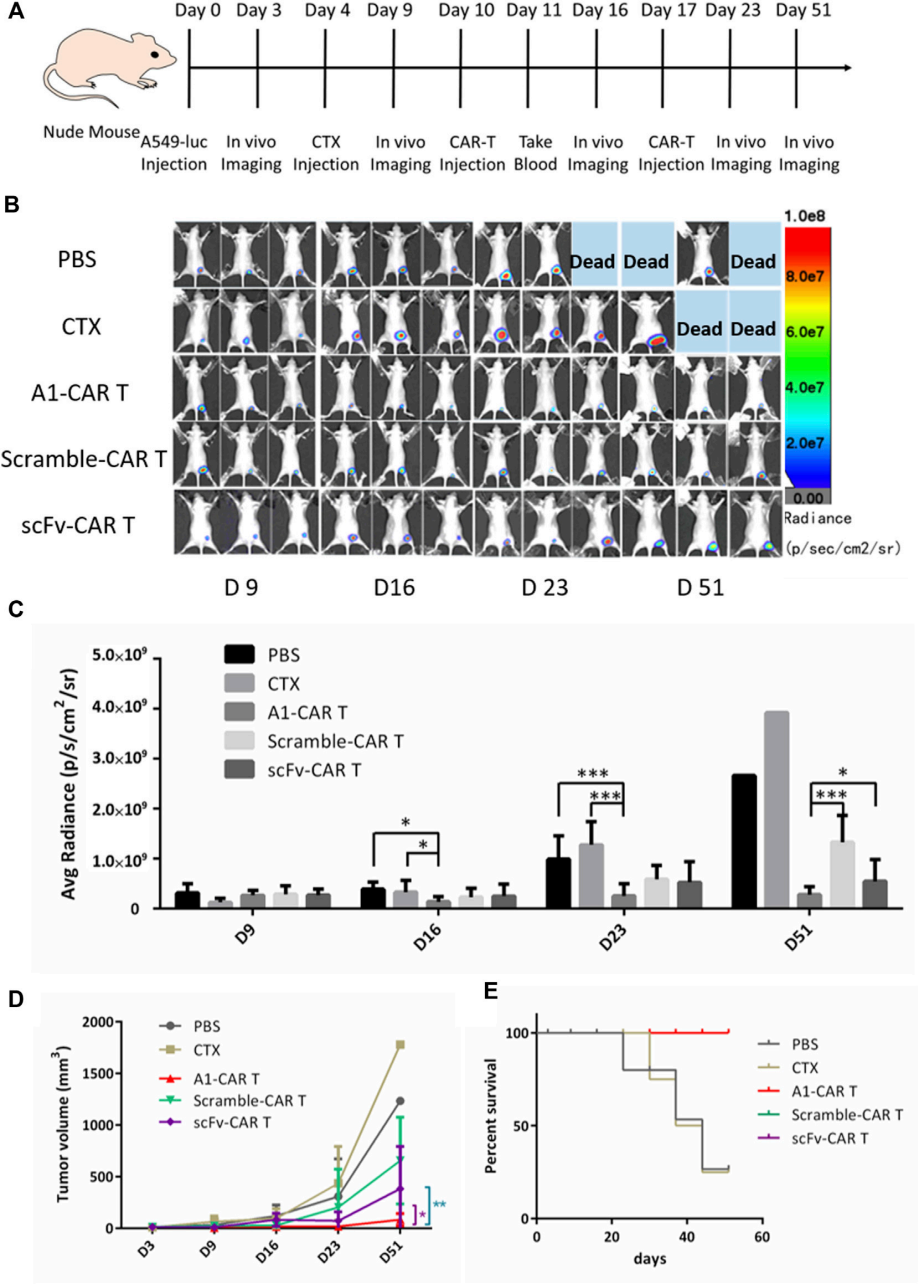
Bispecific CARs are more effective in inhibiting the growth of xenograft tumors. **(A)** The injection process of CARs to the mice with tumor xenografts. **(B)** A1-CARs demonstrated potent antitumor activity indicated by bioluminescent imaging with the IVIS Lumina II system (PerkinElmer) every week for a total of five times **(C)** The bioluminescence intensities in mice were measured. *n* = 4 mice. Values represent mean ± SD. *p* = 0.023 (D16, PBS vs. CTX + A1-CARs); *p* = 0.016 (D16, CTX vs. CTX + A1-CARs); *p* = 3.5 × 10^–4^ (D23, PBS vs. CTX + A1-CARs); *p* = 7.2 × 10^–4^ (D23, CTX only vs. CTX + A1-CARs); *p* = 1.7 × 10^–4^ (D51, CTX + A1-CARs vs. CTX + Scramble-CARs); *p* = 0.03 (CTX + A1-CARs vs. CTX + scFv-CARs). Calculated *p* values from two-sided Student’s t test. n = 4 mice/group. **(D)** The tumor volume average measurements and values of mice are measured. *p = 0.019, **p = 0.009. *n* = 4 mice/group. *T* tests were utilized to decide the *p* values. **(E)** Survival of the mice are shown.

A549-luc is a lung cancer cell line with stable luciferase express. We s. c. inoculated nude mice with A549-luc the human lung carcinoma cells on day 0. Mice were burdened with tumors were treated with 100 μL PBS or 100 μL, 200 mg/kg cyclophosphamide (CTX) on day 4. For CARs treatment in part of cyclophosphamide group, mice were not only injected i. p. with CTX to deplete host lymphocyte compartments on day 4. For CARs treatment, mice were given i. p. injection with 200 mg/kg cyclophosphamide for depleting circulating lymphocytes on the fourth day. After 6 days, 5 × 10 ^6^ of scFv-T cells or peptide-based CARs were given i. v. injection on days 10 and 17. (Figure 3A). For PBS group and CTX group, mice were injected i. v. with 100 μL PBS or CTX. The scramble-CARs was used as negative control. The positive rate of all group CARs was greater than 38% (Figure 1C). Representative bioluminescence images of tumor-burdened mice in each group are shown in Figure 3B. The total brightness (P/s) of each group (Figure 3C) and tumor volume (Figure 3D) were recorded. The ability of inhibit tumor growth of A1-CARs was consistent with what we observed *in vitro*. There are clear differences between these groups. In xenograft mouse models, two doses of A1-CARs inhibited tumor growth. Notably, one of four (25%) of the mice cells were tumor-free after two dosages in group A1-CARs on days 51, but neither in group scFv-CARs group nor in scramble-CARs group. The tumor growth rate in A1-CARs group or scFv-CARs group treated mice was 82.65% or 32.73% lower than that in control scramble-CARs group, respectively (Figure 3C). Mice were euthanized once the tumor size reached 2000 mm^3^. The survival curve also shows the differences in survival for all CARs groups contrast with the PBS and CTX group were statistically significant (*p* < 0.01). And A1-CARs more significantly decreased the tumor growth contrast with Scramble-CARs or scFv-CARs (Figure 3E).

These results confirm that A1-CARs are very effective in suppressing tumor growth.

### Peptide-Based CARs Show Better Cytokine Release and Persistence in Vivo

On day 51, tumor masses were detached, fixed for IHC. And then, Tumor masses were stained by IFN-γ and GZMB. IHC results revealed that the secretion level of IFN-γ and GZMB of A1-CARs group was significantly higher compared to the scramble-CARs group (Figures 3A–C). The area of IFN-γ was 63.6% more in A1-CARs treated tumors more than scFv-CARs treated, 6 times more than the untreated tumors (Figure 5B). As shown in Figure 5C, the area of GZMB was 110.5% more in A1-CARs treated tumors more than scFv-CARs treated, 7 times more than the untreated tumors.

**FIGURE 4:**
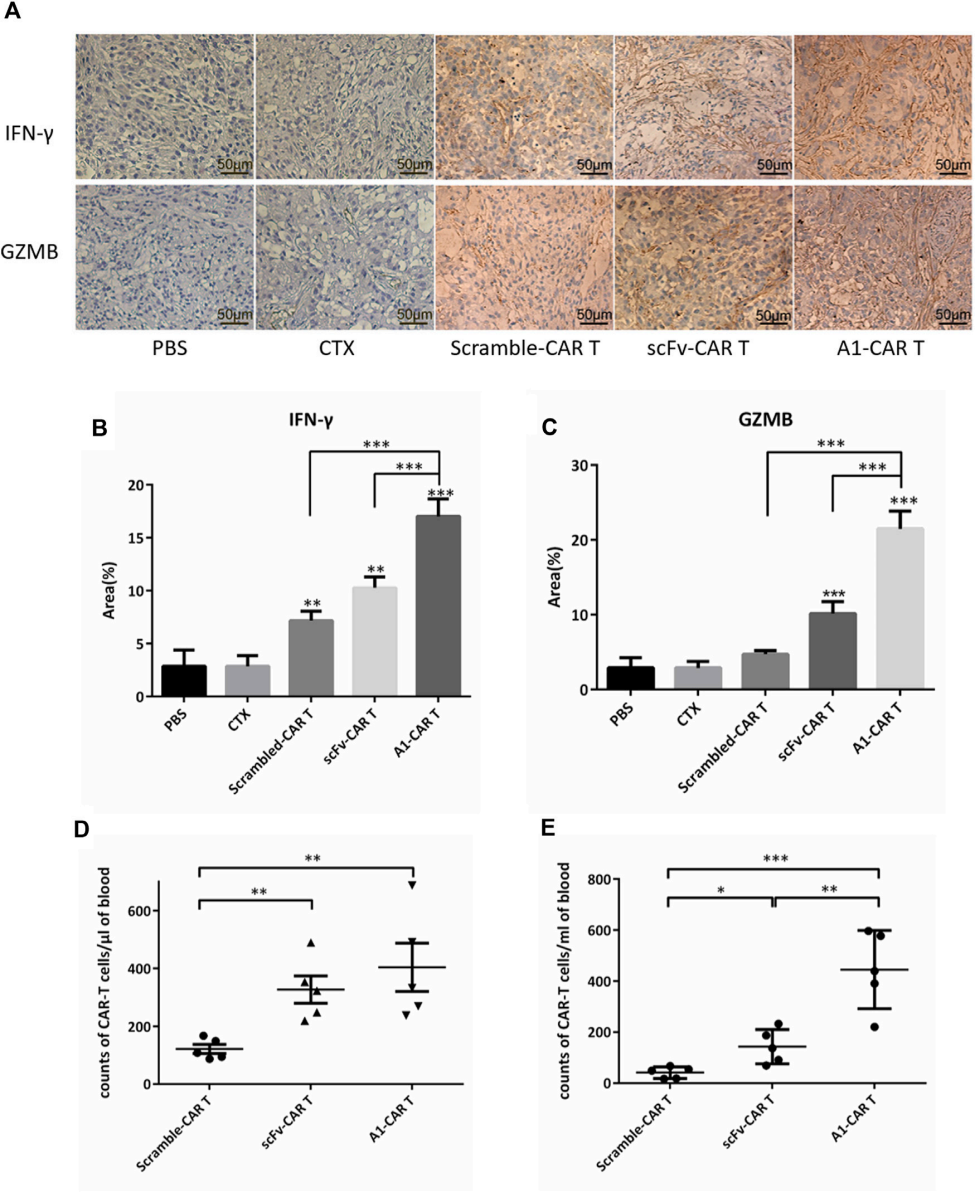
Peptide-based CARs had robust expansion ability and long-term functional persistence *in vivo*. (A) IFN-γ and GZMB immune staining images of tumor tissues from various groups. **(B,C)** Immunohistochemical quantitative analysis of IFN-γ and GZMB. Data were given as mean ± SD from five independent experiments. ***p < 0.001. *T* tests were utilized to decide the *p* values. **(D,E)** After 1 day and 21 days following infusion of CARs, CARs in peripheral blood were quantified by flow cytometry. *n* = 5 mice. Data is shown with mean ± SD. *p < 0.05, **p < 0.01, ***p < 0.001. *T* tests were utilized to decide the *p* values.

To trigger elimination of large tumors, CARs may require robust expansion ability and long-term functional persistence *in vivo*. We evaluated the durability of peptide-based CARs in mice burden malignancies to further verify mechanisms of A1-CARs treatment on tumor progression. Blood was obtained 1 day and 21 days following the first CARs injection. Then, we measured the amount of CD3^+^ T cells. Substantial differences were observed in different groups mice bearing tumor. A1-CARs showed greater persistence at day 31 than scramble-CARs or scFv-CARs. (Figures 4D,E), indicating that small peptide is beneficial for survival of CARs contrast with scFv.

### Treatment With A1-CARs Reduced On-Target, Off-Tumor Toxicity

**FIGURE 5:**
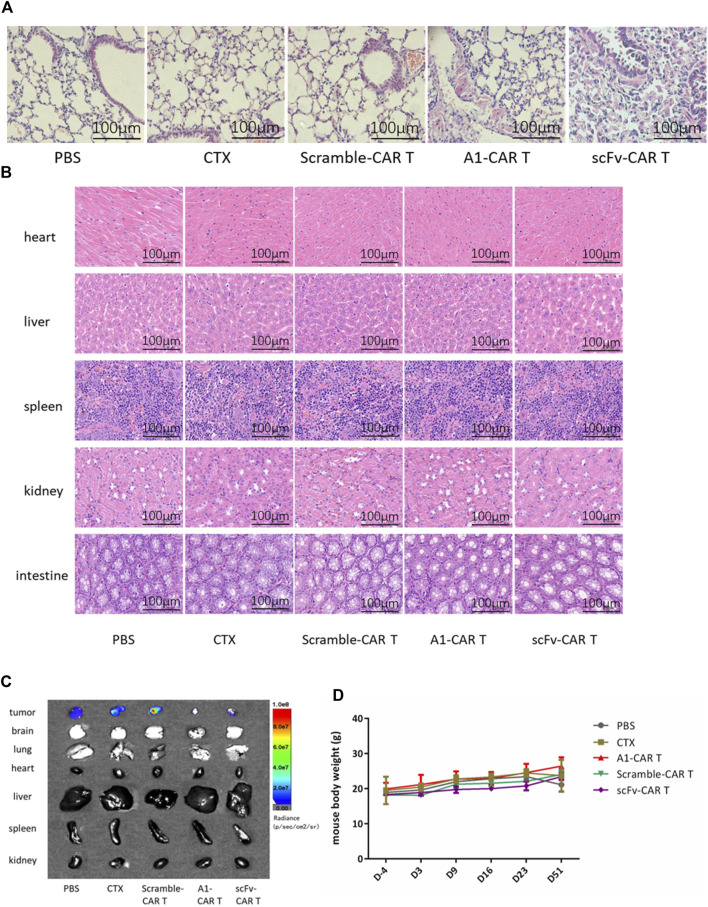
Treatment with A1-CARs reduced on-target, off-tumor toxicity. On day 51, mice were sacrificed after 5 minutes. **(A)** Lung tissue was stained with H&E. **(B)** Heart, liver, spleen, kidney and intestine tissue was stained with H&E. **(C)**On day 51, mice were injected i. p. with D-Luciferin potassium salt and were sacrificed after 5 minutes. Tumor tissues and organs (lung, heart, liver, spleen, kidney, brain and intestine)were resected from the mice, and measured the bioluminescence by the IVIS Lumina II system (PerkinElmer). **(D)** The mice body weight was calculated. *n* = 4 mice. Values represent mean ± SD.

We further investigated the cytotoxicity against normal organs and tissues. As observed in H&E stained tissues, no visible side effects were observed upon repeated administration. The lung tissue of mice in the scFv-CARs group showed obvious fibrosis, with fewer alveoli and denser cells, showing obvious tissue lesions. Unlike the scFv-CARs group, immunogenicity or on-target-off-tumor effect did not adversely affect the lung tissue in A1-CARs group (Figure 5A).

In order to verify whether the second-generation CARs have toxic side effects on other normal organs and tissues, H&E staining was performed and photographed under an upright microscope. As results shown in Figure 5B, the heart, liver, spleen, kidney and intestine of mice showed normal, with no group differences and no evidence of damaged lesions.

Under instant detection by bioluminescence microscopy, the various organs (lung, heart, liver, spleen, kidney, brain and intestine) of the mice showed no tumor metastasis and no significant size differences (Figure 5C). As shown in Figure 5D, the body weights of each group mice were non significantly changing by CARs.

We conclude that peptide was beneficial for generating CARs that are effective and safe in suppressing tumor growth in xenograft mouse model.

## Discussion

Our findings settle one of the governing challenges of CARs therapy for treating solid tumors: the expression of TAA on some organ tissues. In the results, we certified the ability of peptides-based CARs to target the tumor cells *in vitro* assays and be delivered to the interior of solid tumors *in vivo* assays, which indicated that peptide-based CAR would be an alternative treatment for relapsed and refractory solid tumor.

Compared with the great progress of CARs in the treatment of hematological tumors, many innovations are needed for CARs to defeat solid tumors. One of the major difficulties is the lack of unique antigens. Most antigens recommended as CARs focuses to treat solid tumors are selective to particular disease types, and restricted data on antigens for most of solid tumors put numerous cancers far off for CARs treatment (Dotti et al., 2014). To some extent, the effect of CARs depended on the differential articulation of the objective antigen in tumor and normal tissue. Existing information showed that CARs with known serious on track, off-cancer poison can be reengineered by partiality tuning, holding effect *in vivo* while lessening or diminishing poisonousness. Specifically, the 4D5 CAR in view of trastuzumab had deadly poisonousness (Morgan et al., 2010), because of acknowledgment of physiologic degrees of ErbB2 communicated in cardiopulmonary tissues (Gross and Eshhar, 2016). Past work from Chmielewski and Xiaojun Liu (Thomas et al., 2011; Tran et al., 2013; Brudno and Kochenderfer, 2019; Ghorashian et al., 2019) proposed that the high partiality CARs displayed less segregation between target cells with high or low objective articulation levels. Through lessening the KD of scFv utilized in CARs by 2-to 3-log, a significant improvement in the helpful record for ErbB2 and EGFR CARs. Here, compared to the high liking scFv, we exhibited that CARs with peptides showed similarly powerful effect against target cancers.

Despite the target A549 cell was used to confirm this of-idea, this approach maybe apply in the other targets, such as antigen Her-2, FAP and ErbB2 and so on (MacKay et al., 2020). Maybe they can further enhance the activity of antitumor, in spite of that those targets have serious side effects in CARs with single-chain antibodies.

As with therapies combining different checkpoint-blocked inhibitors, combining CARs with other methods is the best choice for solid tumors, such as antibodies, radiation, or small-molecule drugs. The results of the EGFR-targeted and fibrin-fibronectins-targeted CARs demonstrate that the way of peptide-based CAR can be used in a variety of tumors. When peptides with appropriate specificity are recognized, without any modification, they can express by inserting into the backbone of the CAR. The scope of syngeneic tumors that can be targeted by CARs in mouse xenograft model is broadened by building a platform for the production of peptide-based CARs. Due to peptides are easy to express, as antigen recognition domains for CARs, they are attractive (Pameijer et al., 2007; Whilding et al., 2017; Wang et al., 2020).

CARs therapy are remarkable potential to treat cancers (Larson and Maus, 2021). Keishi Adachi’s et al. reported that, in addition to serving as direct antitumor effector cells, CARs can also serve as cellular carriers to transfer immunomodulatory molecules into the tumor microenvironment (Adachi and Tamada, 2018). For improvement of the CARs therapy effect, they developed CARs producing CCL19 and IL-7 to simulate the CARs. This strategy could recruit DCs and T cells to tumor environment, strengthening the treatment effects of CARs toward solid tumors. The combination of immune-regulatory factors with CAR improved the anti-tumor effects of CARs. In our work, the peptide-based CARs were produced and optimized, and their functionalities (cytotoxicity, tumor growth inhibition, *etc*) were verified *in vitro* and *in vivo.* Peptide-based A1-CARs recognizing A549 cells showed ligand specific cytotoxicity, which were efficacious in mouse xenograft model. We plan to further modify these peptides CARs for expression of checkpoint pathway inhibitors, chemokines, and cytokines to enhance the CARs trafficking to tumor tissues and evaluate their anti-tumor functions.

Mouse xenograft models still remain universal for the CARs study (Wilkie et al., 2008; Craddock et al., 2010; Johnson et al., 2015). Although these models enable human tumor and CARs studies, several disadvantages are still encountered. For instance, these models lack intact innate as well as adaptive immunity, and are not capable of depicting the clinical immune potential. Compared with xenograft models, immunocompetent or PDX (patient-derived xenografts) models represent a better option for the evaluation of safety during treatment. Therapeutic strategies without immune depletion are in demand, and endogenous anti-tumor immunity profile is significant in tumor surveillance (Fong et al., 2009). Researchers should pay more attention on combination therapies (e.g. cytokine therapies and checkpoint blockade)for improved treatment effect of solid tumors.

Given that the human lung carcinoma is the most common tumor type, we will determine whether peptide-based CARs therapy could exert influence to diverse other tumor models. Our results endow considerable importance to the clinic. On one hand, this strategy shows capacity to enhance the safety and clinical potential of CARs for validated targets. On the other hand, the applicability of our design can be extended to targets that are not druggable previously with CARs due to on-target toxicity. Beyond doubt, CAR peptides with higher safety profile and efficacy can be designed for various common carcinomas.

Taken together, a safe and effective peptides CAR design is proposed here, and *in vitro* and *in vivo*, T cells treated by peptides CAR lentivirus leads to specific and potent inhibition of the human lung carcinoma A549 cells. These results demonstrate that peptide-based CARs therapeutics might represent an intriguing strategy for the treatment of solid tumors. We propose that the peptide-based CARs might be clinically translated for more solid tumor types.

## Data Availability

The original contributions presented in the study are included in the article/Supplementary Material, further inquiries can be directed to the corresponding authors.
