# Supplementary material for: Reforming the Chimeric Antigen Receptor by Peptide Towards Optimized CAR T Cells With Enhanced Anti-Cancer Potency and Safety
Source: Front Bioeng Biotechnol. 2022 Jun 17;10:928169. doi: 10.3389/fbioe.2022.928169 (PMC9247402; doi:10.3389/fbioe.2022.928169)
Supplement: Supplementary file 1 [file DataSheet1.docx]

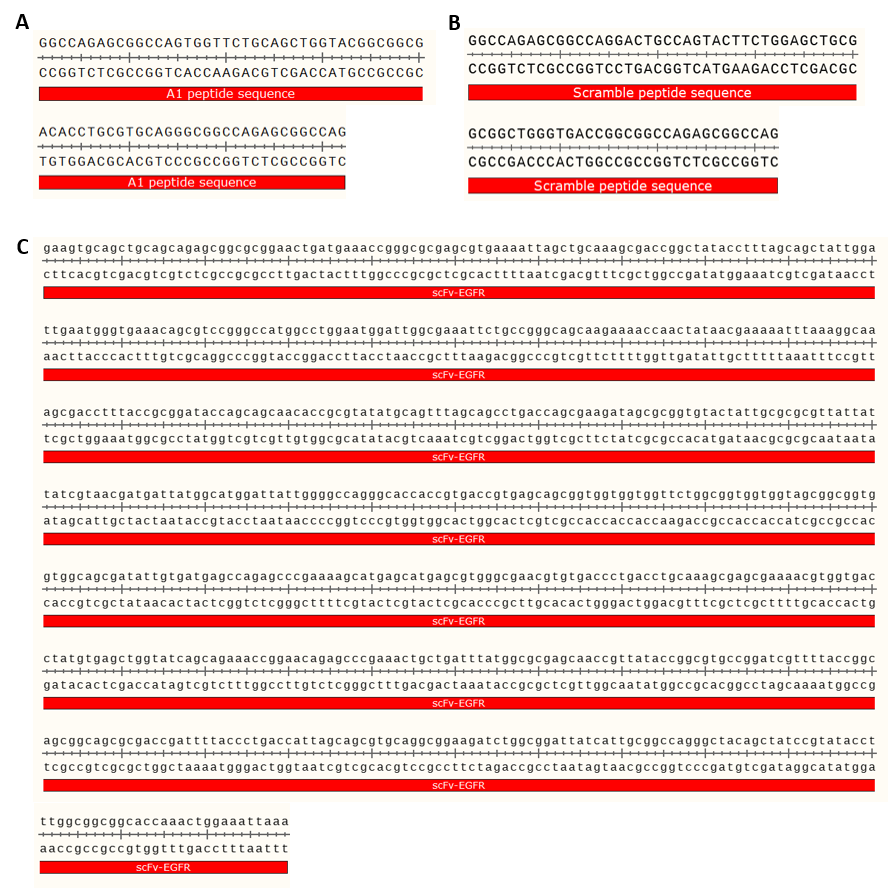


**Supplementary figure 1.** The gene sequence. (A) A1 peptide sequence. (B) Scramble peptide sequence. (C) scFv-EGFR sequence.


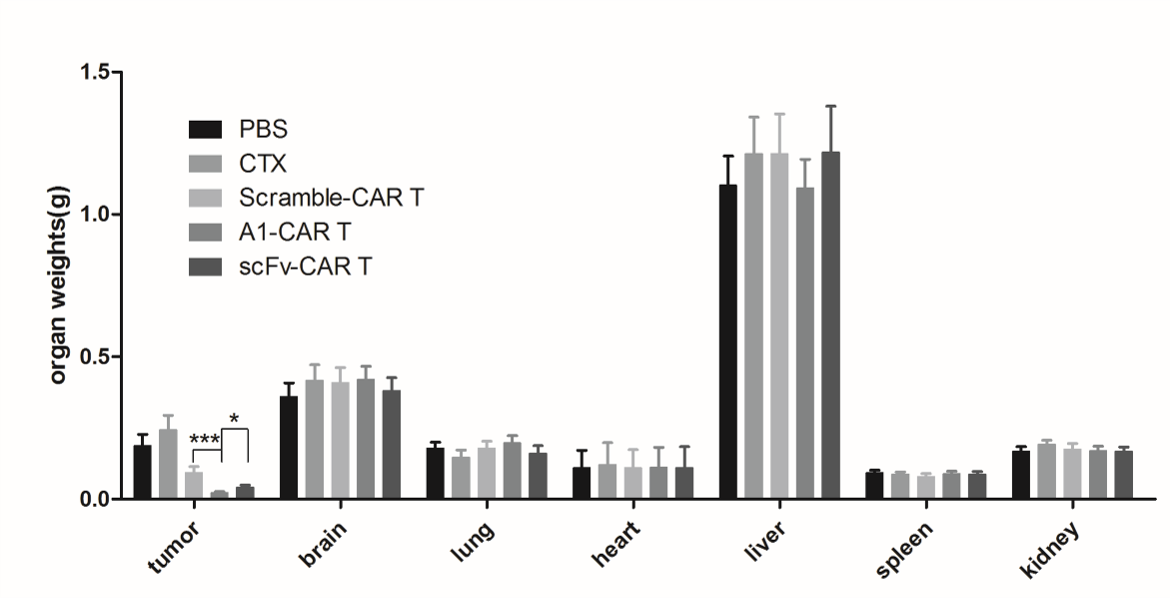


**Supplementary figure 2.** The mice tumor, brain, lung, heart, liver, spleen, kidney weights were calculated. n = 4 mice. P = 5×10^-4^(Scramble-CAR T vs. A1-CAR T);P = 0.02(scFv-CAR T vs. A1-CAR T). Values represent mean ± SD. T tests were utilized to decide the P values. * p<0.05, *** p<0.001.
